# Editorial: Revolutionizing dentistry: the impact of AI and new algorithms on diagnostics, treatment, and patient engagement

**DOI:** 10.3389/fdmed.2026.1834761

**Published:** 2026-04-10

**Authors:** Gustavo Vicentis Oliveira Fernandes, Juliana Campos Hasse Fernandes

**Affiliations:** 1 Peridontics, A. T. Still University – Missouri School of Dentistry and Oral Health, St. Louis, MO, United States; 2 Research Department, GF10 Foundation, St. Louis, MO, United States

**Keywords:** algorithm, artificial intelligence (AI), dentistry, diagnosis, technology, treatment

## Introduction

The integration of algorithms and artificial intelligence (AI) into dentistry marks a transformative era characterized by enhanced diagnostic precision, personalized treatment planning, and improved patient engagement (1). The Research Topic “*Revolutionizing Dentistry: the Impact of AI and New Algorithms on Diagnostics, Treatment, and Patient Engagement*” was conceived to explore these advancements and critically evaluate their implications for clinical practice, research, and education. The contributions in this collection highlight the breadth of innovation across multiple domains of dentistry, reinforcing AI as a pivotal driver of modern oral healthcare.

AI technologies, particularly those based on machine learning and deep learning, have demonstrated remarkable capabilities in analyzing complex dental datasets, including radiographs, clinical images, and patient records. These tools enable earlier detection of pathological conditions, more accurate prognostic assessments, and increasingly individualized treatment strategies. As emphasized in this Research Topic, the convergence of computational power, data availability, and algorithmic sophistication is redefining the boundaries of dental diagnostics and therapeutic decision-making.

One of the central themes emerging from this collection is the expanding role of AI in diagnostics. The systematic review on multimodal large language models for oral lesion diagnosis underscores the growing potential of integrating textual and visual data to enhance diagnostic accuracy. By combining clinical descriptions with imaging inputs, these models represent a significant step toward comprehensive, context-aware diagnostic systems. Their clinical utility lies not only in improving detection rates but also in supporting clinicians in complex decision-making scenarios, particularly in cases involving subtle or ambiguous lesions.

Complementing this perspective, the systematic review published in the present issue, which uses non-invasive quantitative methods to assess blood flow in periodontal and oral soft tissues, highlights the importance of objective, quantifiable diagnostic metrics. While not exclusively AI-driven, many of these technologies are increasingly being integrated with AI algorithms to enable real-time analysis and interpretation. The ability to measure microvascular changes non-invasively has profound implications for monitoring periodontal and peri-implant diseases, offering a pathway to earlier intervention and more precise evaluation of treatment outcomes.

**Figure 1:**
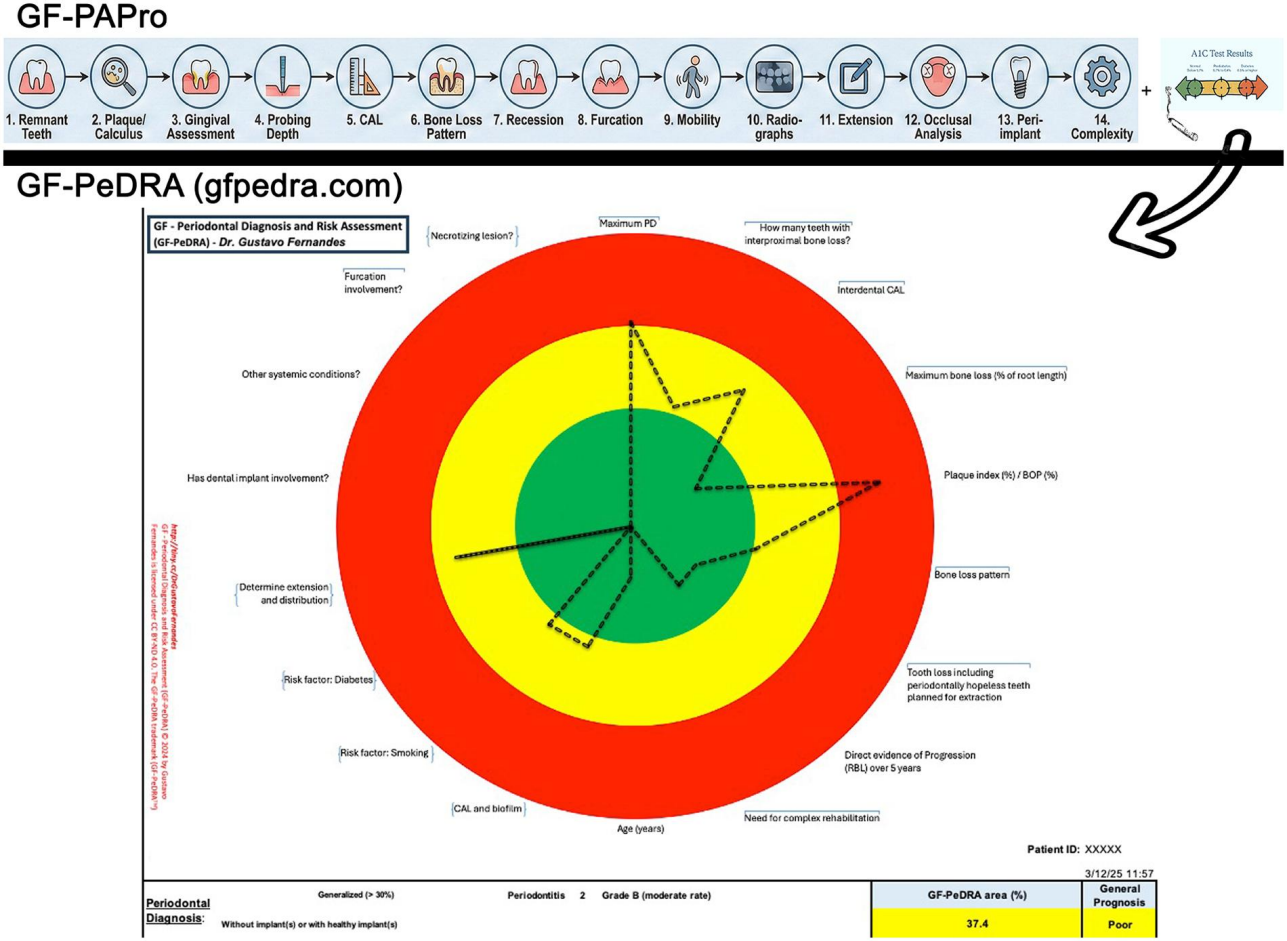
Integration of two algorithm systems to improve the accuracy and precision for periodontal diagnosis. Graphic displaying a periodontal diagnosis workflow, with GF-PAPro (across fourteen assessment steps at the top), followed by a radar/spider chart with colored target zones (green, yellow, red) for risk parameters (GF-PeDRA), resulting in a GF-PeDRA risk assessment of 37.4% (poor prognosis).

In alignment with these advances, the introduction of novel diagnostic tools such as the GF-Periodontal Assessment Protocol (GF-PAPro) (2) and the GF-Periodontal Diagnosis and Risk Assessment (GF-PeDRA – https://www.gfpedra.com) (3) (Figure 1) exemplify the next generation of clinical instruments. These tools are designed to support periodontal and peri-implant assessment through standardized, reproducible metrics and algorithm-driven analysis. By integrating clinical parameters with computational models, they aim to reduce subjectivity, improve diagnostic consistency, and facilitate longitudinal monitoring. Importantly, such innovations reflect the broader trend toward embedding AI directly into clinical workflows, thereby bridging the gap between theoretical development and practical application.

Beyond diagnostics, AI is increasingly influencing treatment planning and execution. The narrative review on AI-driven dynamic orthodontic treatment management illustrates how continuous data integration and predictive modeling can optimize orthodontic care. By enabling personalized progress tracking and adaptive treatment adjustments, AI systems allow clinicians to respond to patient-specific variations in real time. This paradigm shift moves away from static treatment protocols toward dynamic, data-driven strategies that enhance efficiency and outcomes.

Similarly, the exploration of AI in dentistry and dental biomaterials provides insight into how computational approaches are reshaping material science and restorative procedures. AI-assisted design and manufacturing, particularly when integrated with CAD/CAM technologies, enable the production of highly customized dental restorations with improved precision and reduced error rates. These advancements not only enhance clinical performance but also streamline laboratory workflows, contributing to greater overall efficiency in dental practice.

Another notable contribution to this Research Topic is the review of retrieval-augmented generation (RAG) in dentistry. This emerging approach combines large language models with external knowledge retrieval systems, enabling more accurate and contextually relevant information generation. In dental settings, RAG has the potential to support clinical decision-making, patient education, and research synthesis by providing evidence-based, real-time insights. Its application also extends to the development of intelligent virtual assistants and chatbots, which can improve patient engagement by delivering timely information and facilitating communication.

Patient engagement, indeed, represents a critical dimension of AI integration in dentistry. The ability of AI systems to provide personalized education, automate administrative processes, and enhance communication contributes to improved patient adherence and satisfaction. As highlighted in the scope of this Research Topic, tools such as AI-driven chatbots and digital platforms can empower patients with accessible, understandable information about their oral health and treatment plans. This shift toward patient-centered care is essential in promoting preventive strategies and long-term oral health outcomes.

Despite these promising developments, several challenges must be addressed to ensure the responsible and effective implementation of AI in dentistry. Data privacy and security remain paramount concerns, particularly given the sensitive nature of healthcare information. Robust regulatory frameworks and ethical guidelines are necessary to safeguard patient data while enabling innovation. Additionally, the successful integration of AI into clinical practice requires adequate training and education for dental professionals. Bridging the knowledge gap between technological development and clinical application is crucial to maximizing the benefits of AI.

Another important consideration is the need for standardized metrics and benchmarking of AI tools. Variability in data sources, model architectures, and evaluation methods can hinder the comparability and generalizability of findings. Efforts to establish consensus guidelines and validation protocols will be essential in ensuring the reliability and clinical applicability of AI systems.

Collectively, the articles in this Research Topic underscore the multifaceted impact of AI on dentistry, spanning diagnostics, treatment planning, material science, and patient engagement. They highlight not only the technological advancements achieved to date but also the opportunities and challenges that lie ahead. The integration of tools such as GF-PAPro and GF-PeDRA further exemplifies the translational potential of AI-driven innovations, demonstrating how research can directly inform clinical practice.

As dentistry continues to evolve in the digital age, the role of AI will undoubtedly expand, shaping the future of oral healthcare. Interdisciplinary collaboration among clinicians, researchers, engineers, and policymakers will be essential in guiding this transformation. By fostering innovation while addressing ethical and practical considerations, the dental community can harness the full potential of AI to improve patient care and advance the field.

In conclusion, this Research Topic provides a comprehensive overview of the current AI landscape in dentistry, offering valuable insights into its applications and implications. It serves as both a reflection of progress and a call to action for continued exploration and responsible implementation of AI technologies. The future of dentistry is being redefined, and AI stands at the forefront of this revolution.
